# Passion for Violently Themed Music and Psychological Well-Being: A Survey Analysis

**DOI:** 10.3390/bs12120486

**Published:** 2022-11-30

**Authors:** Merrick Powell, Kirk N. Olsen, Robert J. Vallerand, William Forde Thompson

**Affiliations:** 1 School of Psychological Sciences, Macquarie University, Macquarie Park 2109, Australia; 2 Centre for Elite Performance, Expertise, and Training, Macquarie University, Macquarie Park 2109, Australia; 3 Département de Psychologie, Université du Québec à Montréal, P.O. Box 8888, Montreal, QC H3C 3P8, Canada; 4 Faculty of Society and Design, Bond University, Robina 4226, Australia

**Keywords:** psychological well-being, passion, violently themed music, fandom, Dualistic Model of Passion, satisfaction with life, vitality, meaning in life, thriving, depression, anxiety, affective experiences

## Abstract

While the benefits to mood and well-being from passionate engagement with music are well-established, far less is known about the relationship between passion for explicitly violently themed music and psychological well-being. The present study employed the Dualistic Model of Passion to investigate whether harmonious passion (i.e., passionate engagement that is healthily balanced with other life activities) predicts positive music listening experiences and/or psychological well-being in fans of violently themed music. We also investigated whether obsessive passion (i.e., uncontrollable passionate engagement with an activity) predicts negative music listening experiences and/or psychological ill-being. Fans of violently themed music (*N* = 177) completed the passion scale, scale of positive and negative affective experiences, and various psychological well- and ill-being measures. As hypothesised, harmonious passion for violently themed music significantly predicted positive affective experiences which, in turn, predicted psychological well-being. Obsessive passion for violently themed music significantly predicted negative affective experiences which, in turn, predicted ill-being. Findings support the Dualistic Model of Passion, and suggest that even when music engagement includes violent content, adaptive outcomes are often experienced. We propose that the nature of one’s passion for music is more influential in predicting well-being than the content or valence of the lyrical themes.

## 1. Introduction

The positive experiences and adaptive psychosocial functions of engaging with music are well-documented and play a significant role in contributing to fans’ psychological well-being [1,2,3,4]. However, the potential benefits for psychological well-being from passionate engagement with music containing negatively valenced themes are less understood. One type of negatively valenced music commonly investigated in this domain is sad music, or music often characterised by a minor mode, slow tempo, and emotional lyrics [5]. Many fans of sad music report experiencing predominantly positive and pleasurable emotions, alongside the experience of sadness, in response to music [6]. However, fans who are prone to depressive symptoms are susceptible to having predominantly negative emotional outcomes [5,6,7,8]. While sad music has received considerable empirical attention, an emerging body of research on negatively valenced music has focused on the psychosocial risks and benefits of music with violent themes [9]. Themes of violence in music are primarily found within extreme metal and rap subgenres and are characterised by lyrics that explicitly depict horrific actions of violence that include murder, rape, and body mutilation, often perpetrated by men against women [10,11,12].

Music containing violent themes has been the subject of increasing public concern over the last few decades. For example, heavy metal artists have been blamed for motivating shootings and suicides in the 1990s [13]. More recently, drill rap artists have been banned from performing live due to fears of evoking violence in its fans [14,15]. Conversely, research conducted on passionate fans of violently themed music has detailed a very different picture to these concerns. Fans of heavy metal music and rap music with overtly violent lyrics report overwhelmingly positive affective experiences including empowerment, joy, and peacefulness, along with other psychosocial benefits such as mood management, formation and affirmation of identity, and strengthening of social bonds [9,12,16].

This research suggests that the valence or content of the music itself is not a good predictor of listeners’ experience. For example, negatively valenced violent music does not often elicit anger or fear in its fans, and sad music does not always result in sadness or despair [9,17]. Indeed, individual characteristics of music fans and the specific nature of their engagement with music may be more useful predictors of psychosocial outcomes than whether the music is deemed ‘aggressive’, ‘violent’, or ‘melancholic’. To investigate this overarching hypothesis, Olsen, Powell et al. (2022) employed the Dualistic Model of Passion to investigate whether fans’ type of passionate engagement with music containing violent or non-violent themes is an important factor in predicting the likelihood of positive and negative psychosocial outcomes [2].

Initially developed by Vallerand et al. (2003), the Dualistic Model of Passion posits two distinct types of passion that are related to positive and negative outcomes [18]. First, harmonious passion describes engagement with an activity that is without any attached contingencies, meaning that an individual can engage with the activity purely for the positive experiences it affords and can disengage when the activity is unsuitable or undesirable [19,20]. Second, obsessive passion occurs when a person is overly reliant on an activity for their self-esteem or has attached other internal and external pressures to the activity. This leads to a rigid persistence with the activity, whereby engagement uncontrollably continues even when negative outcomes are experienced or expected. Rumination and negative emotions are also experienced when one is unable to engage with the activity, or while doing other activities [21,22].

Drawing from Vallerand et al.’s (2003) Dualistic Model of Passion, Olsen, Powell et al. (2022) observed that harmonious passion predicted positive affective responses such as self-reported wonder, joy, and empowerment in fans of violently themed extreme metal and rap music, as well as passionate fans of non-violently themed classical music [2,18]. Further, obsessive passion predicted negative affective experiences such as sadness, tension, and anger in the same fan groups [2]. These findings show the Dualistic Model of Passion provides a useful framework for understanding and predicting the experience of positive and negative outcomes for fans of both violently themed and non-violently themed music.

Although this approach has been informative for gauging affective outcomes immediately after exposure to music, there is little understanding about the relationship between engagement with violently themed music and broader psychosocial outcomes of music engagement, such as psychological well-being. Furthermore, the role of harmonious and obsessive passion for music containing violent themes in the context of fans’ psychological well-being and ill-being is not yet established. The benefits for psychological well-being from engaging with non-violent music are well-known when the experience of older adults [23], young adults [24], and performers [25] are considered. The present study was designed to fill the gap in understanding regarding the potential positive relationship between psychological well-being and music containing violent themes.

In the domain of violent visual media more generally, there have been concerns about the negative effects of sustained engagement on psychological well-being; for example, the potential for heightened levels of depression and anxiety, as well as desensitisation to violence coupled with increased aggressive cognitions and behaviours [26,27]. For fans of music with violent themes, there is no conclusive evidence of desensitisation to violence from short or long-term music listening, or evidence that fans have higher levels of depression than fans of non-violently themed music [10,28]. Rather, fans who report positive psychosocial outcomes in response to music containing violent themes may instead experience a ‘spiral up’ of well-being, where frequent engagement with a favourite activity or hobby results in consistent positive emotions and broadened perceptual and attentional capacity, which creates a positive ‘spiral’ that facilitates increased psychological well-being [22,29,30].

This ‘spiral up’ hypothesis was recently investigated from a sample of 197 passionate music fans of 40 different genres [3]. Specifically, relationships between passion, affective experiences with music, and psychological well-being and ill-being were analysed. Research in psychological well-being and ill-being suggests that these two constructs should be measured distinctly rather than on opposing ends of the same measure, as the absence of ill-being does not necessarily indicate that a person is thriving or flourishing [22]. Path analyses revealed that harmonious passion significantly predicted positive affective experiences with music, which, in turn, predicted a composite measure of psychological well-being [3]. The composite well-being measure contained the satisfaction with life scale, the subjective vitality scale, the brief inventory of thriving, and the ‘presence’ subscale of the meaning in life questionnaire [31,32,33,34]. This finding supported the notion that well-being can ‘spiral up’ for fans of music experiencing positive affective outcomes. On the other hand, obsessive passion significantly predicted negative affective experiences with music, which, in turn, predicted a composite measure of ill-being consisting of the anxiety and depression subscales of the DASS-21 [35]. This finding provided evidence for a reciprocal ‘spiral down’ relationship between fans’ negative affective experiences with music and maladaptive impacts on psychological well-being.

The main objective of the study was to understand whether the valence of the music content, containing many overtly violent themes, influences the capacity of the music to facilitate psychological well-being outcomes for its fans. By utilizing a similar paradigm to that in Powell et al. (2022) to specifically investigate fans of music with violent themes [3], it can be observed whether fan experiences are similar or different across fans of violently and non-violently themed music. Within fans of violently themed music, we specifically examined whether harmonious passion is a predictor of positive affective experiences and psychological well-being, and whether obsessive passion is a predictor of negative affective experiences and psychological ill-being similar to other music fans, and despite the negative valence of the music.

Based on the previously observed positive experiences reported by fans of violently themed music, we hypothesised that harmonious passion for violently themed music would predict psychological well-being in its fans, observed through a composite measure of satisfaction with life, vitality, meaning in life, and thriving (H1a). Further, obsessive passion for violently themed music should predict psychological ill-being, measured by self-reported depression and anxiety (H1b). It was also predicted that harmonious passion for violently themed music would predict positive affective experiences with music listening (H2a), and obsessive passion for music will predict negative affective experiences (H2b). Finally, harmonious passion should predict positive affective experiences with music which, in turn, should predict psychological well-being (H3a) and obsessive passion should predict negative affective experiences with music which, in turn, should predict psychological ill-being (H3b).

## 2. Materials and Methods

### 2.1. Participants

The 177 participants (105 males, 65 females, 7 identifying as non-binary or preferring not to disclose) were recruited online through fan pages for specific extreme metal and rap genres on social media websites or through the platform Prolific (*n* = 106), as well as through the undergraduate psychology student pool from Macquarie University, Australia (*n* = 71). This range of recruitment methods was necessary because self-identified passionate fans of music containing violent themes are a niche subpopulation, and thus all were needed to obtain a sufficient sample size. Participants’ mean age was 28.0 years (*SD* = 9.2) and reported listening to an average of 23.4 h of music each week (*SD* = 19.6). There were significant differences in the ages of the two samples, with the sample recruited online being significantly older (*M* = 31.9, *SD* = 9.3) than the first year psychology sample (*M* = 22.1, *SD* = 5.2), *t*(175) = 8.07, *p* < 0.001, 95% CI [7.40, 12.19].

Initial regression analyses were conducted to assess whether controlling for age differences had a significant influence on the main regression results. Age did significantly negatively predict anxiety scores when included in the model, *β* = −0.24, *t*(3173) = −3.38, *p* < 0.001, but was not significantly associated with any other dependent variables of interest. Further, age did not influence the relationships between the two types of passion and any dependent variables, such that age’s inclusion did not eliminate any significant associations between either type of passion and the dependent variables, and vice versa. Hence, it was deemed unnecessary to control for age in the main analyses reported below. There were statistically significant differences between the two samples for the measure of thriving (*t*(175) = 2.02, *p* = 0.045, 95% CI [0.07, 5.56]), anxiety (*t*(175) = 2.58, *p* = 0.011, 95% CI [0.42, 3.16]), and harmonious passion (*t*(175) = 2.58, *p* = 0.011, 95% CI [0.58, 4.32]), where the online sample had lower scores on anxiety and thriving and a higher score for harmonious passion compared to the undergraduate sample. There were no other differences between the two samples. As these differences were few and the effect sizes were small, the two groups were combined in the main analyses.

The final sample size of 177 was deemed adequate. That is, the sample was slightly below a commonly suggested ratio of 10 observations per parameter estimated, as there were 21 estimated in the final model [36], but far above the ratio of five observations per parameter that is considered the ‘minimum’ for structural equation modelling [37]. Initially, the sample size was collected to meet the desired ratio of 10 observations per parameter estimated, but the removal of ineligible participants and the addition of parameters due to modification indices meant that this was not achieved. Participants recruited through Prolific were paid approximately $5AUD for their participation and undergraduate psychology students participated for course credit. Online fan forum participants went in the draw to win a $100 voucher.

Participant eligibility required an answer of ‘yes’ to two items, one asking whether they enjoy music that contains violent themes, and one asking whether they identified as a passionate fan of music containing violent themes. Further, they had to report a mean score of four or above on five general passion questions, all on a 7-point Likert scale, with an example question including “Listening to this music is part of who I am”. This criterion has been used in other research including in a study of passionate musicians [25]. Data for participants who did not meet these criteria were removed from the final sample. When asked about the genre of music containing violent themes that they were most passionate about, 16 different genres were reported. The most common were Heavy Metal (*n* = 58), Gangsta Rap (*n* = 41), Death Metal (*n* = 23), Black Metal (*n* = 14), and Drill Rap (*n* = 13). The full list and frequencies of each are presented in Appendix A.

### 2.2. Measures

#### 2.2.1. The Passion Scale

The Passion Scale [18] was used to measure fans’ levels of harmonious and obsessive passion for their favourite genre of music. The harmonious and obsessive subscales each contain six items, in addition to five general passion items described above, all measured on a 7-point Likert scale. Powell et al. (2022) adapted the original items to relate specifically to music, and these adapted items were used in this study [3].

#### 2.2.2. Satisfaction with Life Scale (SWLS)

The SWLS [31] was used to assess fans’ well-being through their degree of satisfaction with life experiences to date. The SWLS contains five items on a 7-point Likert scale ranging from “Do not agree at all” (1) to “Very strongly agree” (7).

#### 2.2.3. Subjective Vitality Scale (SVS)

The SVS [32] was administered to assess fans’ levels of well-being through vitality, a measure of energy one has available to themselves. Responses to the 7-item scale are scored on a 7-point Likert scale ranging from “Not at all true” (1) to “Very true” (7).

#### 2.2.4. Brief Inventory of Thriving (BIT)

The BIT [33] was employed as a holistic measure of fans’ well-being through their perception of positive functioning in life. The scale consists of 10 items and is scored on a 5-point Likert scale, from “Strongly Disagree” (1) to “Strongly Agree” (5).

#### 2.2.5. Meaning in Life Questionnaire (MLQ)

The MLQ [34] was used to measure fans’ subjective belief that their lives contain meaning. As such, only the presence of meaning in life subscale was administered in the present study, with the search for meaning in life deemed not relevant to the study’s research questions. The presence of meaning in life subscale contains five items and is scored using a 7-point Likert scale from “Absolutely Untrue” (1) to “Absolutely True” (7).

#### 2.2.6. The Depression Anxiety Stress Scale (DASS-21)

The DASS-21 [35] was used to measure psychological ill-being. The original measure contains three subscales measuring, depression, anxiety, and stress, but only the depression and anxiety subscales were employed in the present study because they are the two most common measures used to assess ill-being. These two subscales contain seven items each and participants were asked to report how frequently various situations applied to them in the last week, measured on a 4-point Likert scale ranging from “Did not apply to me at all” (0) to “Applied to me very much, or most of the time” (3).

#### 2.2.7. Scale of Positive and Negative Experience (SPANE)

The SPANE [38] measured fans’ positive and negative affective experiences with their favourite music. The scale contains two subscales, with six positive and six negative items, measured on a 5-point Likert scale ranging from “Very Rarely or Never” (1) to “Very Often or Always” (5). Each item is a single-word experiential item, such as joyful (positive subscale). Participants reported how commonly they usually experience each item during and immediately after listening to their favourite music. Total scores for all measures were calculated by summing the individual item scores. Internal consistency scores for each item are presented in Appendix B.

### 2.3. Procedure

Participants signed up for a study about their passion for listening to music containing violent themes. The study was approved by the Macquarie University Human Research Ethics Committee (ethics No. 52022648036259). The study was conducted online during the COVID-19 pandemic using Qualtrics survey software. The survey began with demographic questions regarding gender, age, and the hours of self-selected music fans listen to in an average week. After completing the SWLS, SVS, BIT, MLQ, and DASS-21, participants were instructed to think about their favourite genre of music that: (1) contains violent themes; and (2) they “love and enjoy, identify with, strongly value, and spend a lot of time listening to”. Participants identified this genre from a list of 10 or were free to add their own. Participants then completed the Passion Scale and SPANE in direct response to their nominated genre and were thanked for their participation.

### 2.4. Data Analysis and Preparation

All data analysis was conducted using SPSS 27, except for the path analysis which was performed using SPSS AMOS 27. The method for identifying and correcting outliers was the same as in Powell et al. (2022), where standardised z-scores and Cook’s distance scores were investigated [3]. Although the Cook’s distance was not greater than one for any score, five scores did have z-scores greater than ±3.29 standard deviations from the mean. These were corrected for by assigning new raw values either one unit larger or smaller than the next most extreme score for that variable [39].

There were no notable issues with linearity, homoscedasticity, or multicollinearity. Probability-probability plots and histograms of the residuals did reveal that the anxiety and depression measures were not normally distributed. As in Powell et al. (2022), bias-corrected bootstrapping with 1000 samples was conducted in the regressions with these variables. As the bootstrapped values were not significantly different to the original regression results, the original was reported and interpreted. The statistics for the bootstrapped analysis are presented in Appendix C.

## 3. Results

Table 1 presents the descriptive statistics and correlations between all variables in the study. Multiple regression analyses were conducted with harmonious and obsessive passion as predictor variables and the measures of satisfaction with life, thriving, vitality, presence of meaning in life, depression, and anxiety as the dependent variables in separate analyses. Harmonious passion significantly predicted the presence of meaning in life and thriving measures, but not the vitality or satisfaction with life measures. Obsessive passion significantly predicted both ill-being measures of depression and anxiety. These results, presented in Table 2, partially supported H1a and fully supported H1b.

To test the relationships between passion and affective experiences, multiple regressions were conducted as outlined above, but with positive and negative affective experiences with music as dependent variables instead of the well-being and ill-being measures. Harmonious passion was a significant positive predictor of positive affective experiences and obsessive passion positively predicted negative affective experiences. Further, harmonious passion negatively predicted negative affective experiences. Hence, both H2a and H2b were supported and the results from these analyses are presented in Table 3.

The final hypothesis, investigated through path analysis, was that harmonious passion would positively predict positive affective experiences, which would in turn positively predict psychological well-being (H3a). It was also hypothesised that obsessive passion would positively predict negative affective experiences, which would positively predict psychological ill-being (H3b). A standardised composite measure of well-being was created from the different well-being measures used above, as they used different measurement scales. The ill-being measure was created simply by summing the anxiety and depression scores, as they were measured on the same scale.

A model was created with direct paths between harmonious passion and positive affective experiences, positive affective experiences and psychological well-being, and harmonious passion and psychological well-being. The same paths direct paths were created was the same for obsessive passion, negative affective experiences, and ill-being. There were also paths for the association between positive and negative experiences and the association between well-being and ill-being. Covariance paths were also included for the two types of passion, and for the total hours of music listened to weekly, to control for its influence. Modification indices suggested additional direct paths from hours per week to both psychological well-being and ill-being. Both paths were included in the final model, which revealed an acceptable model fit, *χ*^2^ (df = 7) = 11.62, *p* = 0.114, CFI = 0.99, RMSEA = 0.06, GFI = 0.97, NFI = 0.96.

**Figure 1:**
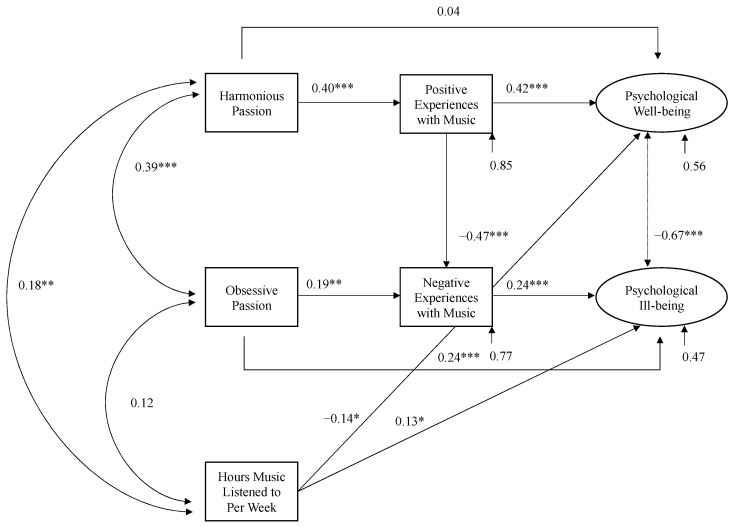
The role of the two types of passion and affective experiences with music on ratings of psychological well-being and ill-being, controlling for hours of music consumed weekly. Numbers in the figure refer to standardised correlation coefficients. *p < 0.05; ** *p* < 0.01; *** *p* < 0.001.

In this final model, all paths were significant, except for the path between harmonious passion and psychological well-being. This suggests that positive affective experiences fully mediated the relationship between harmonious passion for music and psychological well-being in fans of music containing violent themes. As the path between obsessive passion and psychological ill-being remained significant, this suggests that negative affective experiences partially moderated the relationship between obsessive passion for music and psychological ill-being in fans of music containing violent themes. The full model is depicted in Figure 1.

## 4. Discussion

The objective of the study was to examine whether harmonious passion for music containing violent themes would predict positive music listening experiences and psychological well-being for its fans, and whether obsessive passion would predict negative music listening experiences and psychological ill-being. The study built on a previous study that surveyed passionate fans of 40 different genres in which the above relationships were observed [3]. The present study sought to understand whether such relationships would be observed despite the negative valence of the music. Results revealed that for fans of violently themed music, harmonious passion positively predicted two of the well-being measures employed in the study, the measures of presence of meaning in life and thriving, but not satisfaction in life or vitality. Harmonious passion also positively predicted positive affective experiences with music, and negatively predicted negative affective experiences with music. Obsessive passion positively predicted the ill-being measures of anxiety and depression, as well as negative affective experiences with music.

Path analyses investigated the relationship between all these variables and revealed that, as hypothesised, harmonious passion predicted positive affective experiences, which predicted a composite measure of psychological well-being. Positive affective experiences fully mediated the relationship between harmonious passion and psychological well-being, as the indirect path between the two was not significant. Further, obsessive passion predicted negative affective experiences, which predicted a composite measure of psychological ill-being. Negative affective experiences partially mediated the relationship between obsessive passion and psychological ill-being, as the indirect path between the two was still significant.

The findings of the present study reveal that harmoniously passionate fans of violently themed music have positive experiences with such music, and that these positive experiences significantly contribute to aspects of their psychological well-being. Such findings reflect the notion of ‘spiral up’ well-being, where frequently experienced positive affective responses to music can create consistent positive experiences that can contribute to one’s psychological well-being [30]. This supports previous findings that fans experience overwhelmingly positive emotional and affective responses to violently themed music [2,12] and extends these findings to show that such experiences contribute to overall psychological well-being. Further, these findings reflect those in the Powell et al. (2022) study on a broad sample of passionate music fans [3]. Thus, even though the content of this music may contain themes of violence, assault, and torture, fans can and do experience adaptive psychosocial outcomes from their passionate engagement, as long as they exhibit harmonious passion.

On the other hand, obsessive passion for violently themed music predicted maladaptive outcomes, including psychological ill-being. Continued negative affective experiences resulting from obsessive passion partially contribute to symptoms of anxiety and depression, suggesting a ‘spiral down’ ill-being relationship from continued negative affective experiences with music. This outcome may help to explain reports of ruminative cycles and other maladaptive types of music engagement; for example, where people intend to use music to regulate their negative moods but end up feeling worse [40]. In a music therapy or clinical setting, understanding a fan’s level of obsessive passion is an important application of these findings, especially if fans report using music as a means of regulating affect. Measuring obsessive passion may be a crucial step in breaking ruminative cycles. An awareness of the impact of obsessive passion by clinicians may lead to effective strategies to help clients regulate their moods without compounding rumination tendencies. Negative affective experiences only partially mediated the relationship between obsessive passion and psychological ill-being, meaning that there may be factors behind the ‘spiral down’ that were not captured in the present study. One potential contributor may be the ruminative and other negative experiences obsessive passionate fans have when they are unable to engage with their favourite music, as past research has observed in various activities [22]. Future research is required to establish whether this applies to passionate music fans.

The present results support the notion that the type of passionate engagement with music appears more influential to the outcomes of music engagement than the valence of the music itself. Previous research has observed positive associations between harmonious passion and adaptive or protective outcomes for non-music activities that commonly hold negative public perceptions and have potentially negative outcomes, including gambling and computer gaming [41,42]. Furthermore, obsessive passion has been associated with maladaptive outcomes, such as increased negative affect, in activities commonly regarded as universally positive, such as yoga [43]. As the present study is the first investigation to establish relationships between passion and well-being for passionate listeners of violently themed music, future research could support and extend the present findings in meaningful ways. Specifically, the design of the study was cross-sectional. Thus, employing a longitudinal design can help increase the validity of conclusions when causal links between the development of passion for such music and its influence on psychological well-being are investigated.

Two unexpected results were observed in the current set of findings. First, harmonious passion positively predicted the measures of presence of meaning in life and thriving, but not satisfaction with life or vitality. Although relationships between harmonious passion and psychological well-being were observed when all well-being measures were combined, these non-significant relationships between harmonious passion and satisfaction with life or vitality suggest that there may be some specific nuanced aspects of well-being associated with passion for violently themed music. Specifically, satisfaction with life is proposed to be a measure of hedonic well-being, referring to the pursuit of pleasure and avoidance of pain [44]. The other three measures are proposed to be measures of eudaimonic well-being, or the pursuit and experience of meaning and purpose in life [45]. It may be the case that passion for violently themed music is not as strongly associated with hedonic well-being, as part of the fan experience involves being engaged with experiences that reflect pain or difficult emotions [12]. Regarding vitality, it is possible that harmonious passion does not facilitate experiences of energy and alertness specifically but still leads to experiences of meaning, purpose, and value in life as observed by relationships with the other two eudaimonic measures. Further investigation would be required to replicate and confirm these results and proposed explanations.

The second unexpected finding were the positive predictive path between hours per week of music consumed and psychological ill-being and the negative predictive path between hours per week of music consumed and psychological well-being. Hours of music consumed per week was not significantly associated with obsessive passion and was positively associated with harmonious passion. Therefore, the positive relationship between hours of music consumed per week and psychological ill-being may suggest that there is another mechanism or trait that predicts negative outcomes from excessive consumption of music containing violent themes once passion is controlled for. One possibility is to measure a greater range of maladaptive listening traits using the Healthy Unhealthy Music Scale [46]. Future research could use this scale to see if maladaptive listening traits such as worsening mood and reconnecting to bad memories are associated with greater hours of music consumed per week, as it may be indicative of ruminative cycles of negative listening in which maladaptive engagement is sustained for long periods. Such ongoing maladaptive engagement may also explain the presence of the negative association between weekly music consumption and psychological well-being when harmonious passion is controlled for.

The present study contained some limitations. Firstly, the final sample size was slightly below the desired number for the path analysis conducted. In targeting a niche group of fans with specific eligibility requirements, our final sample of 177 was deemed sufficient to address our primary hypotheses but did not reach the suggested sample size of 210 identified by previous research and expert guidance for models of this kind [36]. Future research using larger samples should ideally be undertaken, especially to verify the path analysis results and to strengthen confidence in the validity of the present findings. Further, the study was cross-sectional. To understand the capacity for fandom to contribute to longer-term well-being or increases in well-being over time, a longitudinal design would be required. For example, a study could investigate if harmonious passion for music containing violent themes at certain time points predicts well-being at later time points to further corroborate the findings of the present study. The study also used financial incentives to recruit many participants. Financial incentivization is effective in facilitating increased participation [47]. However, it has been observed that participants are more likely to lie about their eligibility for participation when greater financial incentives are offered [48]. This effect may be stronger in those of a lower socioeconomic status [49]. While the financial remuneration in this study was small relative to medical research payments, such incentivization may have led to some people overreporting their fandom of music containing violent themes. Future research may also seek to gather broader demographic information from participants, such as their socioeconomic status and level of education, to understand the potential impacts of these variables on the relationships observed in the present study. The role of cognitive reappraisal and its relationship with harmonious passion and positive outcomes from passionate engagement presents a promising line of future inquiry, as has been observed in passionate athletes [20].

To conclude, the present study supports the growing body of evidence that harmonious passion for an activity positively contributes to one’s psychological well-being, even when the content of the activity contains violent or aversive negatively valenced themes, in this case, music with violent themes. Results detailed that harmonious passion was a significant predictor of positive affective experiences and psychological well-being. Specifically, harmonious passion predicted positive affective experiences with music, which, in turn, predicted psychological well-being and fully mediated the original relationship between harmonious passion and well-being. Thus, harmoniously passionate fans of music with violent themes have an adaptive relationship with this music, whereby they can flexibly engage with the music in order to experience positive affect, while disengaging with the music when negative outcomes are expected or apparent. In contrast, obsessive passion predicted negative affective experiences and psychological ill-being, with obsessive passion partially mediating the relationship between obsessive passion and ill-being. These findings were similar to those observed for fans of a range of music genres in a previous study, supporting the notion that the type of passionate engagement a person has with music appears to contribute more to the outcomes of their music engagement than the style or genre of the music. These findings provide empirical evidence to public and Governmental debates regarding the censorship, sales and performance rights of music containing violent themes, while also highlighting the potential psychosocial vulnerabilities of obsessively passionate music fans for clinical contexts.

## Figures and Tables

**Table 1:** Descriptive Statistics and Correlations for All Variables in the Study.

Variable	*M*	*SD*	1	2	3	4	5	6	7	8	9	10
1. Harmonious Passion	27.71	6.28										
2. Obsessive Passion	17.45	7.38	0.39 **									
3. Satisfaction with Life	19.56	7.42	0.11	0.11								
4. Meaning in Life—Presence	20.75	7.71	0.27 **	0.07	0.61 **							
5. Thriving	34.23	9.14	0.17 *	0.03	0.78 **	0.77 **						
6. Vitality	28.16	9.49	0.12	0.04	0.69 **	0.65 **	0.80 **					
7. Anxiety	4.66	4.57	0.05	0.33 **	−0.24 **	−0.26 **	−0.31 **	−0.31 **				
8. Depression	7.03	6.19	−0.05	0.16 *	−0.62 **	−0.61 **	−0.72 **	−0.72 **	0.60 *			
9. Positive Experiences	22.93	3.96	0.40 **	0.07	0.39 **	0.41 **	0.51 **	0.43 **	−0.11	−0.33 **		
10. Negative Experiences	11.35	4.10	−0.22 **	0.16 *	−0.26 **	−0.24 **	−0.35 **	−0.23 **	0.37 **	0.37 **	−0.46 **	
11. Hours Per Week of Music	23.37	19.55	0.18 *	0.12 *	−0.20 **	−0.12	−0.17 *	−0.14	0.21 **	0.21 **	0.03	<0.01

Note. * *p* ≤ 0.05; ** *p* < 0.01.

**Table 2:** Harmonious Passion and Obsessive Passion as Predictors of Measures of Well-Being and Ill-Being.

Construct	Scale Item	HP	OP	*R* ^2^
Passion Type				
	Harmonious Passion	-	-	-
	Obsessive Passion	0.39 ***	-	-
Psychological Well-being				
	Satisfaction with Life	0.08	0.08	0.02
	Meaning in life—Presence	0.29 ***	−0.04	0.08
	Thriving	0.19 *	−0.05	0.03
	Vitality	0.13	−0.01	0.02
Psychological Ill-being				
	Anxiety	−0.04	0.32 ***	0.10
	Depression	−0.06	0.20 *	0.03

Note. * *p* ≤ 0.05; *** *p* < 0.001. Coefficients are standardised beta weights from multiple regression analyses except for the association between the two types of passion, which is a Pearson correlation coefficient (r).

**Table 3:** Harmonious Passion and Obsessive Passion as Predictors of Experiences with Music.

Construct	Scale Item	HP	OP	*R* ^2^
Passion Type				
	Harmonious Passion	-	-	-
	Obsessive Passion	0.39 ***	-	-
Affective Experiences with Music				
	Positive Experiences	0.45 ***	−0.11	0.17
	Negative Experiences	−0.33 ***	0.29 ***	0.12

Note. *** *p* < 0.001. Coefficients are standardised beta weights from multiple regression analyses except for the association between the two types of passion, which is a Pearson correlation coefficient (r).

**Figure A1:**
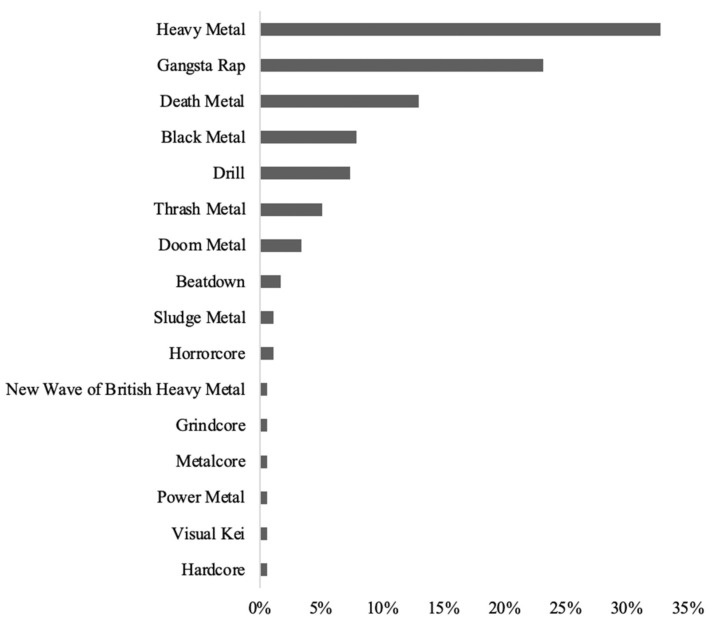
List of genres selected as favourite and most passionate about by participants. Percentages refer to the proportion of participants who selected each genre as their favourite.

## Data Availability

The data presented in this study are openly available through OSF at https://osf.io/jgkhe/.

## Appendix B

**Table A1:** Internal Consistency Coefficients for Each Measure.

Scale Set	Scale Item	*α*
Passion Scale		
	HP	0.80
	OP	0.81
Satisfaction with Life Scale		
	Satisfaction with Life	0.90
Meaning in Life Questionnaire		
	Presence	0.92
Brief Inventory of Thriving		
	Thriving	0.93
Subjective Vitality Scale		
	Vitality	0.91
Depression Anxiety Stress Scale		
	Depression	0.94
	Anxiety	0.87
Scale of Positive and Negative Experience		
	Positive Experience	0.85
	Negative Experience	0.83

## Appendix C

**Table A2:** Results of the Bootstrapped Regression Analyses that Violated Assumptions.

Construct	Scale Item	*b* HP	*SE* HP	*b* OP	*SE* OP
Psychological Ill-being					
	Anxiety	−0.07 (−0.17, 0.02)	0.08	0.23 *** (0.15, 0.31)	0.05
	Depression	−0.14 (−0.30, 0.00)	0.08	0.18 ** (0.05, 0.30)	0.07

Note. ** *p* < 0.01; *** *p* < 0.001; *b* = unstandardised beta coefficients from bootstrapped multiple regression, with 95% bias corrected and accelerated confidence intervals presented in brackets. *SE* = standard errors of the unstandardised beta coefficients. Age was entered as a predictor to account for the significant age difference between the two different populations.
